# Proof of concept of a multimodal intravital molecular imaging system for tumour transpathology investigation

**DOI:** 10.1007/s00259-021-05574-y

**Published:** 2021-10-15

**Authors:** Zhen Liu, Tao Cheng, Stephan Düwel, Ziying Jian, Geoffrey J. Topping, Katja Steiger, Qian Wang, Rickmer Braren, Sybille Reder, Markus Mittelhäuser, Christian Hundshammer, Benedikt Feuerecker, Sung-Cheng Huang, Markus Schwaiger, Franz Schilling, Sibylle I. Ziegler, Kuangyu Shi

**Affiliations:** 1 Department of Nuclear Medicine, School of Medicine, Technische Universität München, Munich, Germany; 2 Institute of Biomedical Engineering, Shenzhen Bay Laboratory, Shenzhen, China; 3 Department of Pathology, School of Medicine, Technische Universität München, Munich, Germany; 4 Department of Radiology, Technische Universität München, Munich, Germany; 5 Department of Molecular and Medical Pharmacology, David Geffen School of Medicine, University of California, Los Angeles, USA; 6 Department of Nuclear Medicine, University Hospital LMU Munich, Munich, Germany; 7 Department of Nuclear Medicine, University of Bern, Bern, Switzerland; 8 Department of Informatics, Technische Universität München, Munich, Germany

**Keywords:** Intravital imaging, Multimodal imaging, Window chamber, Tumour microenvironment, Transpathology, Positron imaging, Fiducial marker, Glycolysis imaging

## Abstract

**Background:**

Transpathology highlights the interpretation of the underlying physiology behind molecular imaging. However, it remains challenging due to the discrepancies between in vivo and in vitro measurements and difficulties of precise co-registration between trans-scaled images. This study aims to develop a multimodal intravital molecular imaging (MIMI) system as a tool for in vivo tumour transpathology investigation.

**Methods:**

The proposed MIMI system integrates high-resolution positron imaging, magnetic resonance imaging (MRI) and microscopic imaging on a dorsal skin window chamber on an athymic nude rat. The window chamber frame was designed to be compatible with multimodal imaging and its fiducial markers were customized for precise physical alignment among modalities. The co-registration accuracy was evaluated based on phantoms with thin catheters. For proof of concept, tumour models of the human colorectal adenocarcinoma cell line HT-29 were imaged. The tissue within the window chamber was sectioned, fixed and haematoxylin–eosin (HE) stained for comparison with multimodal in vivo imaging.

**Results:**

The final MIMI system had a maximum field of view (FOV) of 18 mm × 18 mm. Using the fiducial markers and the tubing phantom, the co-registration errors are 0.18 ± 0.27 mm between MRI and positron imaging, 0.19 ± 0.22 mm between positron imaging and microscopic imaging and 0.15 ± 0.27 mm between MRI and microscopic imaging. A pilot test demonstrated that the MIMI system provides an integrative visualization of the tumour anatomy, vasculatures and metabolism of the in vivo tumour microenvironment, which was consistent with ex vivo pathology.

**Conclusions:**

The established multimodal intravital imaging system provided a co-registered in vivo platform for trans-scale and transparent investigation of the underlying pathology behind imaging, which has the potential to enhance the translation of molecular imaging.

**Supplementary Information:**

The online version contains supplementary material available at 10.1007/s00259-021-05574-y.

## Introduction

**Fig. 1:**
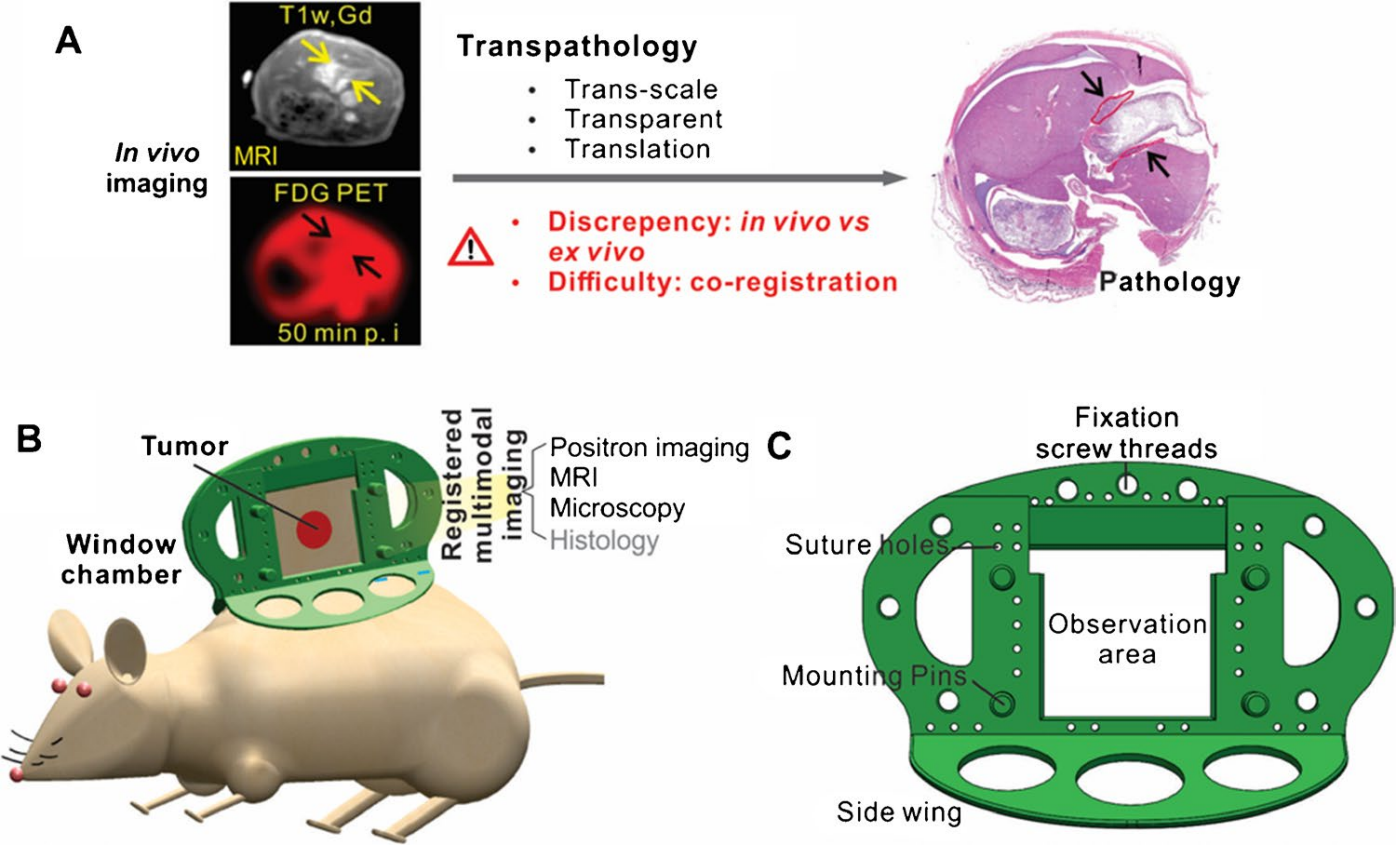
**A** The concept of transpathology research and its challenges; **B** a concept of integrative MIMI system based on an animal model with a dorsal skin window chamber for transpathology investigation, which enables positron imaging, MRI and optical imaging within the same intact framework to allow better co-registration; **C** a detailed sketch of the designed dorsal skin window chamber with mounting pins to fix a positron camera and optical coordinate system, fixation screw threads and suture holes as further fiducial markers for localization with imaging such as MRI

Transpathology is a new theory to summarize the endeavour of molecular imaging for deciphering underlying pathophysiology [1, 2], which consists of trans-scale, transparent and translational investigation of imaging signals (Fig. 1A). It holds the great potential to transparentize tissue and better present the underlying pathophysiological information, as well as to better facilitate the translational processes from the bench to the bedside. However, the practice of transpathology is complicated by discrepancies between in vivo imaging and ex vivo pathology. Conventionally, tissues need to be sampled or resected for the investigation by microscopy [3–6]. Although these in vitro methods have been widely used in various applications, they are destructive and have limited ability to provide insight into in vivo dynamics [7]. Another crucial problem for the comparison between imaging and pathology is the co-registration of in vivo and ex vivo measurements. It is difficult to find useful landmarks or similarities between the images with such a huge resolution difference and typical co-registration algorithms are not applicable [8]. Also, the complicated preparation procedure of the histology sections introduces substantial distortions, and it is thus almost impossible to localize the physiological features precisely in conventional imaging methods.

Intravital imaging is a powerful tissue investigation method to provide high-resolution information of the tissue microenvironment in an intact animal [9, 10]. Different transparent window models have been developed to investigate various types of tumours. Chronic window models such as dorsal skin windows and cranial windows expose a stable tumour microenvironment and allow longitudinal observation of tumour evolution [11, 12]. Intravital imaging can identify in vivo tissue morphological and metabolic characteristics like *pH* or *pO*_*2*_, using various investigation methods such as fluorescence microscopy [13–15] and phosphorescence lifetime imaging [16, 17]. The integration of clinical imaging such as MRI or PET with intravital imaging facilitates the identification of detailed physiological features of imaging elements directly from the same intact tissue [18, 19]. In that study, a dedicated surface receiver coil was applied to obtain high-quality MRI [18]. With careful design of the dynamic protocol, DCE-MRI was acquired to quantify the blood perfusion of the tissue [18]. In another study, direct positron imaging was proposed to improve the sensitivity and resolution of PET imaging within a window chamber [19].

The aim of this study is to establish a multimodal intravital molecular imaging (MIMI) system to support the investigation of transpathology (Fig. 1B). It is based on animal model with a dorsal skin window chamber, which is compatible with the high-resolution MRI, positron imaging and microscopic imaging and which allows precise co-registrations. A pilot test of the multimodal trans-scale imaging and the co-registration accuracy was performed.

## Materials and methods

### Dorsal skin window chamber for multimodal intravital imaging

For transpathology research, a MIMI system is proposed to integrate positron imaging, MRI and optical imaging (e.g. microscope) within an intact and rigid frame (Fig. 1B). This rigid frame (Fig. 1C) is established using a dorsal skin window chamber, which is compatible with the abovementioned imaging modalities, including fiducial markers for positioning and localization. The chamber is made from polyetheretherketone (PEEK), which is rigid but flexible, and is chemically inert to most organic and inorganic chemicals and solvents [18]. The mounting pins can be used to fix a positron camera accurately within the chamber. The fixation screw threads and suture holes provide additional fiducial markers for localization with imaging such as MRI, fluorescence imaging and positron imaging.

### Window chamber implantation and tumour transplantation

Athymic RNU nude rats (Crl: NIH-Foxn1rnu, Charles River) were employed for the in vivo test of the MIMI system. The implantation of the multimodal compatible dorsal skin window chamber onto the RNU rat followed the literature [20, 21] with some adaptions. During the chamber implantation, rats were anaesthetized with inhalation of isoflurane (2% in oxygen, 2 L/min). A single-cell suspension of the human colorectal adenocarcinoma cell line HT29 (2 × 10^6^ cells in 0.05 ml PBS) was prepared for subcutaneous injection onto the fascia side of the intact skin layer within the window area. All animal studies were approved by the local government committee for animal protection and welfare (Tierschutzbehörde, Regierung von Oberbayern, with licence protocol number 18–13).

### Positron imaging

A positron camera with a physical resolution of 230 μm was employed for high-resolution 2D imaging within the window chamber area [22, 23]. The window chamber with intact skin side was fixed to an adapter (Supplemental Fig. 2), and the glass side was then mounted to the positron camera via the above-mentioned mounting pins (Supplemental Fig. 4). The cover glass was removed before mounting to the positron camera. A Mylar sheet (6 μm) was placed on the surface of the camera to prevent possible contact with tissue fluid. Positron imaging of 10 min was acquired at 50 min post-injection of ca.150 kBq/g body weight of [^18^F]FDG under anaesthesia.

### MR imaging

The MR images were scanned with a 7 T preclinical MRI system (Discovery MR901, Agilent Technologies, Santa Clara, USA). The rat was fixed on the MR bed, with air heating during anaesthesia and monitoring of the heart rate by electrocardiogram, breathing rate with a pressure-sensitive pad (RAPID Biomedical GmbH), and body temperature. A single-channel flexible radiofrequency (RF) receiver coil (diameter 30 mm, RAPID Biomedical GmbH) was placed on top of the window chamber. A quadrature birdcage resonator (72-mm inner diameter, RAPID Biomedical GmbH) was used for RF transmission. T1-weighted images using a gradient recalled echo sequence (TR = 300 ms, TE = 1.1 ms, 192 × 192 matrix, 1 mm slice thickness, 4 cm × 4 cm FOV, number of excitations (NEX) = 4, flip angle 60°) and T2-weighted images using a fast spin-echo sequence (TR = 3000 ms, TE = 32.7 ms, 192 × 192 matrix, 1 mm slice thickness, 4 cm × 4 cm FOV, NEX = 4, flip angle 90°) were acquired after positioning. Dynamic contrast-enhanced (DCE) MRI was acquired during the injection of Gd-DTPA solution (0.25 mmol/kg) with a flow rate of 160 ml/h using a syringe pump (Pump Elite 11, Harvard Apparatus, Cambridge, USA). The DCE-MRI was acquired using the TRICKS sequence (time-resolved imaging of contrast kinetics) [24]. TRICKS acquisition was started 15 s before the start of the pump injection. The parameters were TR = 4 ms, TE = 1.7 ms, 192 × 192 matrix, 1 mm slice thickness, 4 cm × 4 cm FOV, flip angle 40°. Fifty-six phases were obtained with 28 images in each phase. Each phase measured with a temporal resolution of 3 s. After the DCE-MRI, another T1-weighted MRI scan was acquired with the same the parameters as before the contrast agent injection.

### Optical imaging

Pathophysiological information was observed with a fluorescence microscope (Imager. M2, Zeiss, Germany) equipped with a black/white charge-coupled device (CCD) camera (AxioCam MRm). Fluorescein isothiocyanate (FITC)-dextran (Sigma Aldrich, St. Louis, MO, USA) was administrated to measure perfusion under MMF (midazolam/medetomidine/fentanyl with a concentration of 5/1/0.05 mg/ml) anaesthesia. The window chamber was fixed onto the adapter (Supplemental Fig. 2) and then fixed to the fluorescence microscope. A transparent coordinate plate (Supplemental Fig. 3) was mounted onto the window chamber for localization. A fluorescence image of 1.25 × magnification with a green fluorescent protein (GFP) filter was recorded.

### Phantom test

To assess the co-registration accuracy for different imaging modalities, phantoms were prepared with two or three thin catheters (I.D.: 0.3 mm, O.D.: 0.6 mm) fixed independently within the window chamber using glue and transparent tape. Phantoms were imaged with positron imaging, MRI and fluorescence microscopy. The catheters were filled with [^18^F]FDG (1 MBq/ml) for positron imaging, saline during T1-weighted MRI imaging and FITC-dextran during fluorescence imaging.

### Ex vivo* histological staining*

After imaging, the rat was euthanized by administration of 0.5 ml pentobarbital sodium via tail vein injection under anaesthesia (Narcoren, 16 g/100 ml, Merial GmbH, Germany). The skin along the window area was cut and fixed with 4% para-formalin solution for 1 day, then sunk into 70% ethanol for 2 days. The fixed tissue block was embedded in paraffin and cut for haematoxylin–eosin (H&E) staining. The whole stained tissue slide was recorded by transmission light using a fluorescence microscopy (BZ-9000, KEYENCE). Due to the limited FOV of the microscopy, the tissue within the window area cannot be covered within one microscopic image. Therefore, the tissue slide was recorded one by one using a 4 × objective lens. The images were further combined into a new image with a larger FOV.

### Data processing

All the primary data from different imaging modalities were aligned via the fiducial markers using PMOD (PMOD Technologies Ltd., Zürich, Switzerland). For phantom measurements, the skeletons of the catheters were extracted by estimating the ridgelines of the imaging signals of the catheters with Gaussian function as medial function [25]. The errors of the co-registrations (i.e. the distances between the skeleton lines obtained from the corresponding imaging modalities) were estimated by comparing the skeleton lines.

## Results

### Accuracy of image co-registration from phantom test

**Fig. 2:**
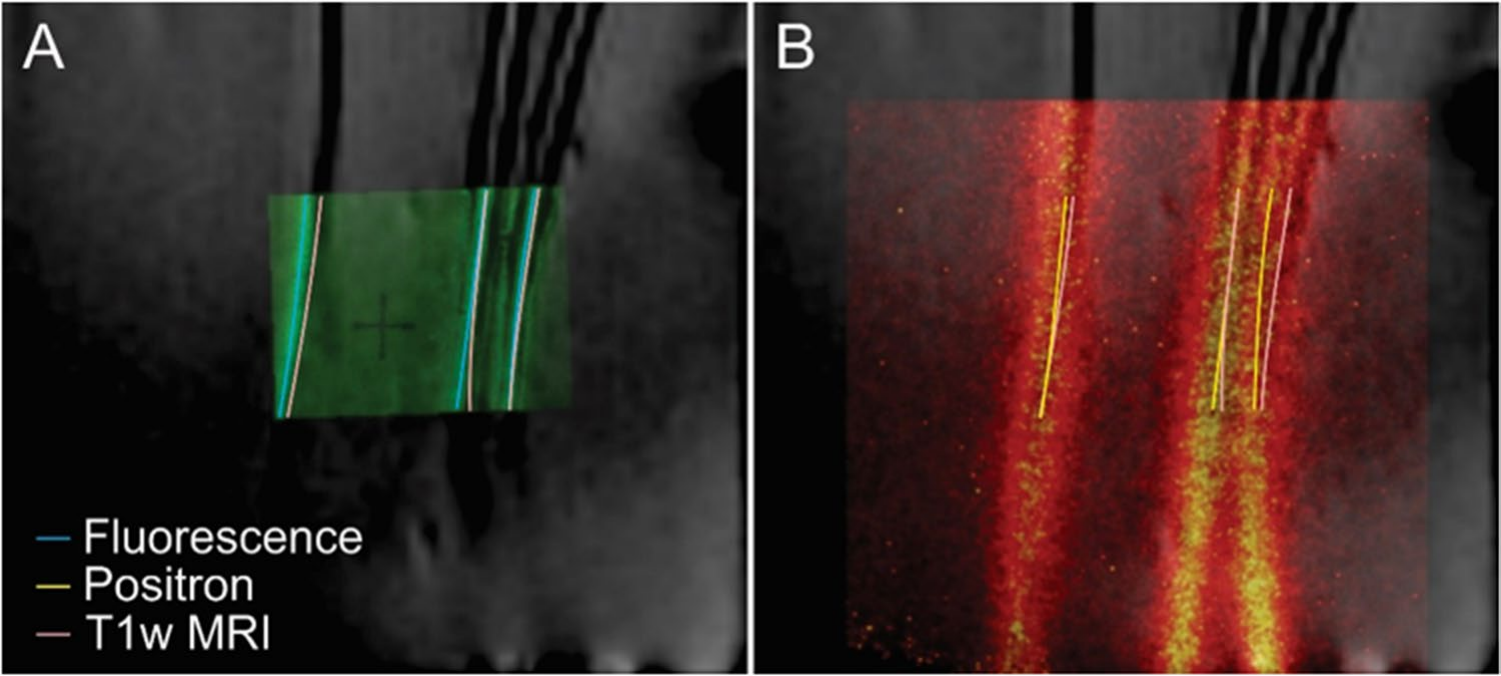
An exemplary fused image of MRI, positron and fluorescence imaging of a phantom with three catheters. The catheters were first filled with [^18^F]FDG for positron imaging, then filled with FITC-dextran for fluorescence imaging and finally filled with saline for MRI imaging. **A** fusion of fluorescence imaging with T1w MRI; **B** fusion of positron imaging with T1w MRI. The lines of different colours denote the skeletons of signals of the catheters in the corresponding imaging modalities

**Fig. 3:**
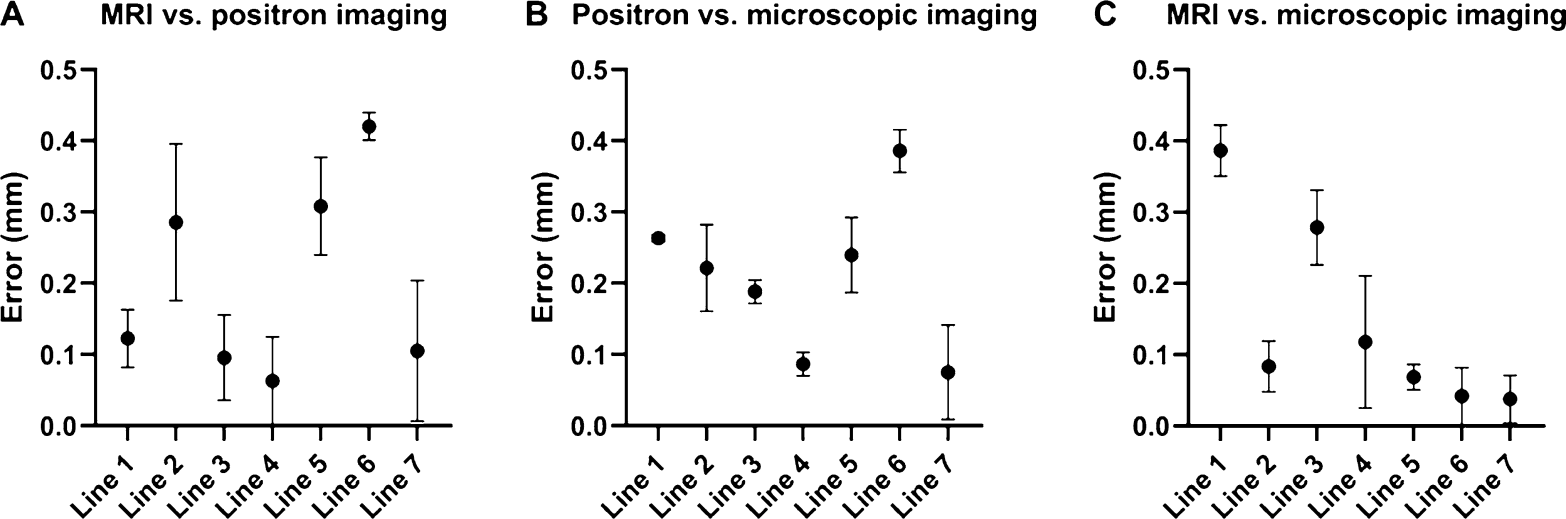
The co-registration position error between the three imaging modalities of all imaged catheters (skeleton lines 1–7). The absolute distance between pairs of points along the skeleton lines is plotted for each line, and the mean and SD are plotted. **A** MRI and positron images; **B** positron and microscopy images; **C** MRI and microscopy images

Images from MRI, positron imaging and fluorescence imaging were aligned via the fiducial markers. Example phantom images are displayed in Fig. 2, with skeleton lines of different modalities extracted from each phantom tube presented on the MRI image. The registration errors between three imaging modalities are plotted in Fig. 3, with statistics for 7 skeleton lines (from three phantoms and the corresponding multimodal imaging experiments) presented in each comparison plot. The co-registration errors were 0.18 ± 0.27 mm between MRI and positron imaging, 0.19 ± 0.22 mm between positron imaging and microscopic imaging and 0.15 ± 0.27 mm between MRI and microscopic imaging.

### Integrated tumour microenvironment imaging

**Fig. 4:**
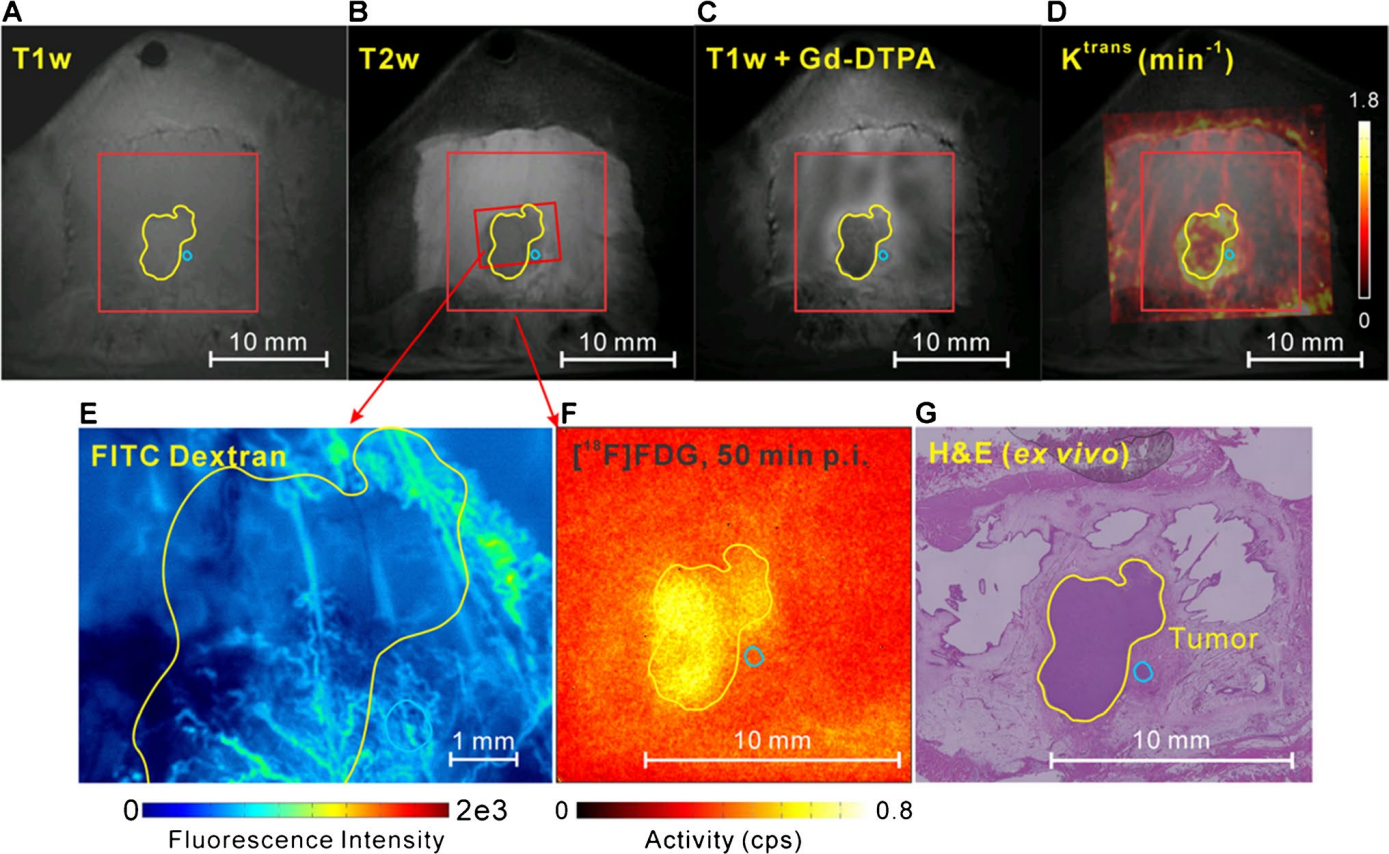
Example imaging results of the multimodal intravital molecular imaging system: **A** a T1-weighted MR image, **B** a T2-weighted MR image, **C** a T1-weighted MR image after Gd-DTPA injection, **D** the K^trans^ map of the MR image; **E** a FITC-Dextran fluorescence image, **F** an [^18^F]FDG positron image and **G** an H&E staining slice of the tumour and periphery tissue. For **E** and **F**, the locations of the images were delineated with the red boxes in **B** and linked with red arrows. Yellow and blue outlines depict the tumour locations in the tissue initially from histology images and then transferred to other measurements. Note that the H&E image was manually co-registered to the in vivo images without the support fiducial markers

Example results of multimodal imaging of a rat with the window chamber are depicted in Fig. 4. The tumour (6.1 mm × 4.9 mm in length and width) in the pre-contrast injection T1-weighted image was brighter compared with the surrounding tissue (Fig. 4A) and darker in the T2-weighted image (Fig. 4B). The T1-weighted MRI image after Gd-DTPA injection depicted the enhanced contrast of the tissue within the window chamber (Fig. 4C). The border of the tumour was clearer after the contrast agent injection. The K^trans^ map of the MR image denoting the blood flow and vascular permeability (influx rate for the contrast agent into the extravascular extracellular space tissue from blood) is presented in Fig. 4D. A fluorescence image (FITC-dextran, Fig. 4E) of the window chamber visualizes the blood perfusion around the tumour tissue. The FITC-dextran fluorescence image of a smaller FOV inside the tumour showed a detailed blood vessel network in the micrometre resolution range. The complexity of the tumour vascular network shown in the fluorescence image was consistent with the MRI observation. From the positron imaging (Fig. 4F), the tumour had generally positive [^18^F]FDG uptake. From the H&E staining image (Fig. 4G), two tumour parts were visualized. A small part of the tumour, indicated with a blue circle in Fig. 4G, was negative in positron imaging.

These three imaging modalities have trans-scaled imaging resolution. The spatial resolution of the MRI, fluorescence image and positron imaging was 156 μm (pixel size), 5.16 μm and 230 μm, respectively. The precise co-registration among these images was accomplished with the aid of the transparent plastic plate with reference pattern (refer to supplemental Fig. 3). Both the fluorescence and positron images can be precisely localized within the MRI image with the aid of the fiducial markers on the window chamber (red box in Fig. 4B). The location of the positron image is always fixed with reference to the four fixation pins, as the positron camera was mounted onto the window chamber with four holes around its detector.

## Discussion

### *Co-registration between *in vivo* and *ex vivo* imaging*

Pathology is considered the gold standard for disease diagnosis. However, it is a sample and test process, facing several limitations, such as invasiveness, incomprehensiveness due to biopsy sampling sites and sampling time, and missing in vivo changes due to limited sampling times. Imaging is a non-invasive tool for intravital observation of pathological changes. The concept of transpathology to linking the imaging with pathology faces two major obstacles for precise evaluation: (1) The discrepancies of scale and resolution between in vivo imaging and ex vivo pathology and (2) the precise co-registration among various trans-scaled imaging modalities and ex vivo pathology. Puri et al. proposed a method for accurate registration of PET images and histopathology slices [8]. They used sea urchin spines as fiducial markers for pathology and ex vivo CT imaging to localize the histopathology slices. The registration error between histopathology and ex vivo CT was reported as 0.86 ± 0.41 mm. The ex vivo CT images were then aligned to the in vivo PET images and the overall registration error between PET and histopathology was 3.0 ± 0.7 mm. In contrast to the registration via ex vivo anatomical imaging, the MIMI system employs a window chamber and assisting holders to immobilize the tissues during the imaging of different modalities. Fiducial markers were designed for the physical localization of the positron imaging and MRI. The localization of microscopic imaging is more difficult due to large differences in the field of view (FOV) and resolution. A transparent plastic reference mounted to the fiducial markers with oriented coordination assists the alignment of the microscopic images. The microscopic imaging is very sensitive to the tilting of the object. The special holder of the window chamber during microscopic imaging ensured that the window chamber was placed parallel to the microscopy’s lens to avoid imaging blurring. These special designs of the MIMI system led to much smaller co-registration errors of 0.18 ± 0.27 mm between MRI and positron imaging, 0.19 ± 0.22 mm between positron imaging and microscopic imaging and 0.15 ± 0.27 mm between MRI and microscopic imaging. The immobilized imaging and the fiducial coordination of the MIMI system facilitate accurate trans-scale image co-registration.

### Multimodal molecular imaging

The combination of PET and MRI provides multimodal clinical or preclinical imaging to visualize anatomical, functional and metabolic features to support the optimization of diagnosis and therapy [26–29]. However, the increased complexity of overlapping or contradictory signal between different modalities within the same tumour due to intratumoural heterogeneity poses substantial challenges to the investigation of underlying physiology [30, 31]. The integration of PET, MRI and intravital imaging in the MIMI system allows fingerprinting the physiological heterogeneity behind multimodal imaging for the same intact tissue [18, 19] and is therefore complementary to conventional preclinical PET/MR in the investigation. The quasi-2D imaging on the intravital window allows higher resolution than conventional 3D PET or MR imaging. With surface coils, it is possible to focus on the thin skin tissue for imaging with a relatively high sensitivity and resolution (< 200 μm) [18]. For the imaging of PET tracers, conventional 3D preclinical scanners are not optimal for imaging of thin tissue within the observation window due to low intrinsic resolution and partial volume effects (PVE) [32]. The positron camera has an intrinsically higher resolution of 230 μm for 2D imaging [22, 23], which is better than the intrinsic resolution of typical 3D preclinical PET scanners [33–35]. In practice, positron imaging is not perfect 2D imaging either and measures the superposed signals from tissues close to the contacting surface. The positron imaging has only spill-in from tissues below the surface, while conventional 3D preclinical PET can have both spill-out and spill-in to the imaging surface. The sensitivity of the positron camera is depth dependent. The spill-in decreases as the depth increases, with half at a depth of 235 μm and vanishes at a depth of 470 μm [36]. In contrast, the spill-in for a high-resolution 3D preclinical PET with 1 mm intrinsic resolution can be 80% from tissue at a depth of 235 μm and 50% at a depth of 470 μm, according to theoretical estimation [37, 38]. Therefore, the proposed positron imaging is more appropriate for quasi-2D imaging of the window chamber.

### Limitations of the MIMI system

The proposed MIMI system has some limitations. (1) Although the fiducial marker facilitated accuracy co-registration for the 2D area within the observation window, it cannot localize the depth of the microscopic image precisely (the Z-axis that perpendicular to the 2D window area). (2) The registration of the ex vivo histological images and in vivo MR images of the MIMI system is still not perfect. We fixed the tissues within the window chamber with PFA initially together with the chamber during the preparation of the histology. However, some distortions and deformations happened during the demounting of the window chamber frames and during the cutting of the tissues, leading to difficulties in preserving the positions of fiducial markers. Therefore, the co-registration of ex vivo histological images and in vivo MR images still needs manual corrections in practice. (3) We used a small magnification of the microscopy in order to cover a large FOV of the tumour to allow the investigation of co-registration to in vivo imaging in this study. However, the large FOV introduced more spill-in artefacts of fluorescence imaging due to increased depth of field. (4) The current study demonstrated proof of concept of the multimodal intravital imaging system; no in-depth physiological investigation has been performed. The partial discrepancy between H&E and positron imaging was not investigated. (5) Although the animals were treated with anti-inflammatory medication after the surgery, unspecific inflammatory [^18^F]FDG uptake cannot be excluded [39, 40]. (6) Here, we made a quantitative assessment about the co-registration accuracy to compare imaging modalities. However, there are other factors that might impair correlation, such as field inhomogeneities in MRI, and this was not evaluated in the study. (7) Although the MIMI system has potential for longitudinal imaging, we did not yet verify this potential.

## Conclusions

A MIMI system was developed for tumour transpathology. The system provides an imaging tool combining three imaging modalities (positron imaging, MRI, fluorescence imaging). The accurate co-registration of the images obtained from the multi-scale imaging modalities ensures a comprehensive view of the tumour microenvironment. This system can bridge the discrepancies between macroscopic and microscopic images and between in vivo and in vitro images and provides a tool for the regional investigation and longitudinal observation of the underlying physiology within intact tumour tissues.

## Supplementary Information

Below is the link to the electronic supplementary material.

Supplementary file1 (PDF 615 KB)

## Data Availability

The datasets used and/or analyzed during the current study are available from the corresponding author on reasonable request.

## Abbreviations

[^18^F]FDG: 2-deoxy-2-(^18^F)fluoro-d-glucose
AFN: Atipamezole/flumazenil/naloxone
BLI: Bioluminescence imaging
CCD: Charge-coupled device
DCE-MRI: Dynamic contrast-enhanced magnetic resonance imaging
FITC-dextran: Fluorescein isothiocyanate-dextran
Gd-DTPA: Gadolinium-diethylenetriamine penta-acetic acid
GFP: Green fluorescent protein
H&E: Haematoxylin–eosin
MIMI: Multimodal intravital molecular imaging
MMF: Midazolam/medetomidine/fentanyl
MR: Magnetic resonance
MRI: Magnetic resonance imaging
NEX: Number of excitation
NIR: Near infrared
PD: Pharmacodynamics
PEEK: Polyetheretherketone
PET: Positron emission tomography
p.i.: Post-injection
PK: Pharmacokinetics
RF: Radio frequency
s.c.: Subcutaneously
TRICKS: Time-resolved imaging of contrast kinetics
