# Supplementary material for: Proof of concept of a multimodal intravital molecular imaging system for tumour transpathology investigation
Source: Eur J Nucl Med Mol Imaging. 2021 Oct 15;49(4):1157–65. doi: 10.1007/s00259-021-05574-y (PMC8921117; doi:10.1007/s00259-021-05574-y)
Supplement: Supplementary file 1 — Supplementary file1 (PDF 615 KB) [file 259_2021_5574_MOESM1_ESM.pdf]

Proof of concept of a multimodal intravital molecular imaging system for tumour transpathology investigation

Authors: Zhen Liu[1,2], Tao Cheng[1], Stephan Düwel[1], Ziying Jian[1], Geoffrey J. Topping[1], Katja Steiger[3], Qian Wang[1], Rickmer Braren[4], Sybille Reder[1], Markus Mittelhäuser[1], Christian Hundshammer[1], Benedikt Feuerecker[1], Sung-Cheng Huang[5], Markus Schwaiger[1], Franz Schilling[1], Sibylle I. Ziegler[1,6], Kuangyu Shi[1,7,8]

Author information

[1] Department of Nuclear Medicine, School of Medicine, Technische Universität München, Munich, Germany

[2] Institute of Biomedical Engineering, Shenzhen Bay Laboratory, Shenzhen, China

[3] Department of Pathology, School of Medicine, Technische Universität München, Munich, Germany

[4] Department of Radiology, Technische Universität München, Munich, Germany

[5] Department of Molecular and Medical Pharmacology, David Geffen School of Medicine, University of California, Los Angeles, USA

[6] Department of Nuclear Medicine, University Hospital LMU Munich, Germany

[7] Department of Nuclear Medicine, University of Bern, Switzerland

[8] Department of Informatics, Technische Universität München, Munich, Germany

***Corresponding author:**

Prof. Kuangyu Shi

Dept. Informatics

Technical University of Munich

Email: k.shi@tum.de

Rat with a multimodal imaging compatible window chamber

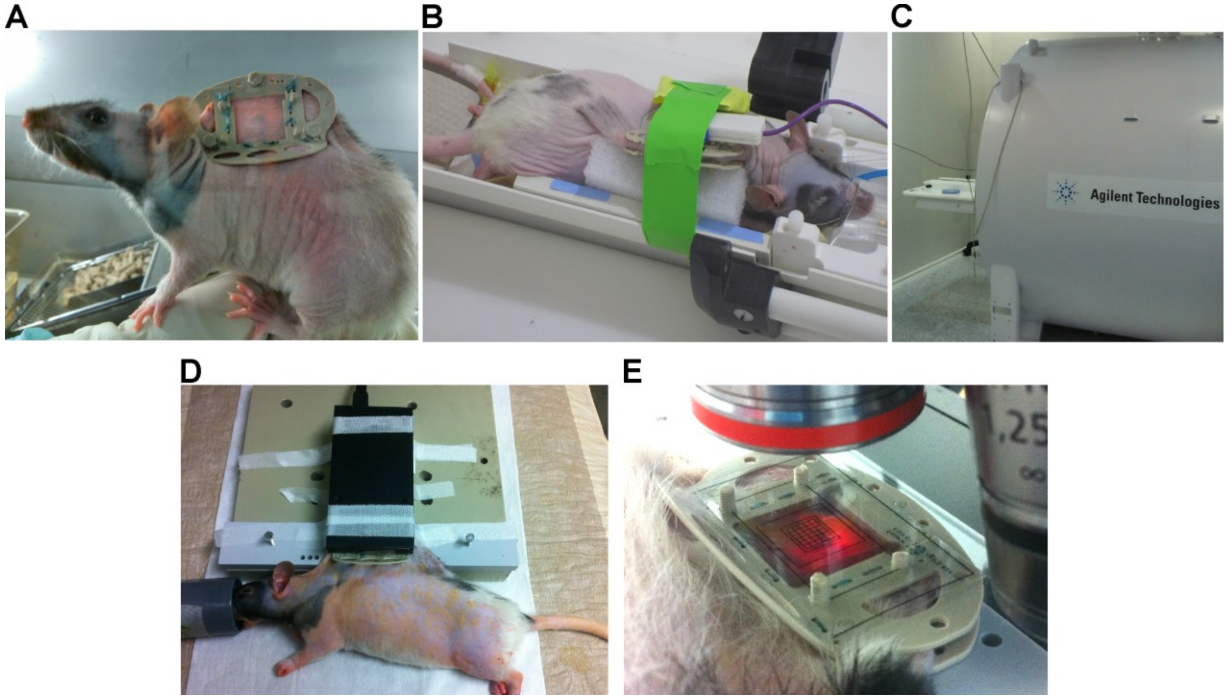

Supplemental Fig. 1 Photos of multimodal compatible rat window chamber tumour model: (A) a rat with the multimodal compatible window chamber; (B) Rat placed on bed prior to insertion into the MR magnet bore, with RF receiver coil affixed to the chamber window; (C) 7 T small animal magnet side view, with animal preparation area at left; (D) the positron imaging setting; (E) the fluorescence imaging setting.

As depicted in Supplemental Fig. 1, the multimodal imaging compatible dorsal skin window chamber was implanted into an RNU rat with ideal compatibility. The RNU rat with the window chamber can move freely in the cage and no abnormal behaviors (such as claw or bite the chamber) were observed. HT-29 tumour cells were successfully transplanted into the skin within the window area. The tumour volume was visible and could be measured with a ruler from day 4 after the transplantation. The window chamber cannot be maintained for longer than ~2 weeks after tumour transplantation because the tumour grows too large. Supplemental Fig. 1B-F show the use of the window chamber for the different imaging modalities.

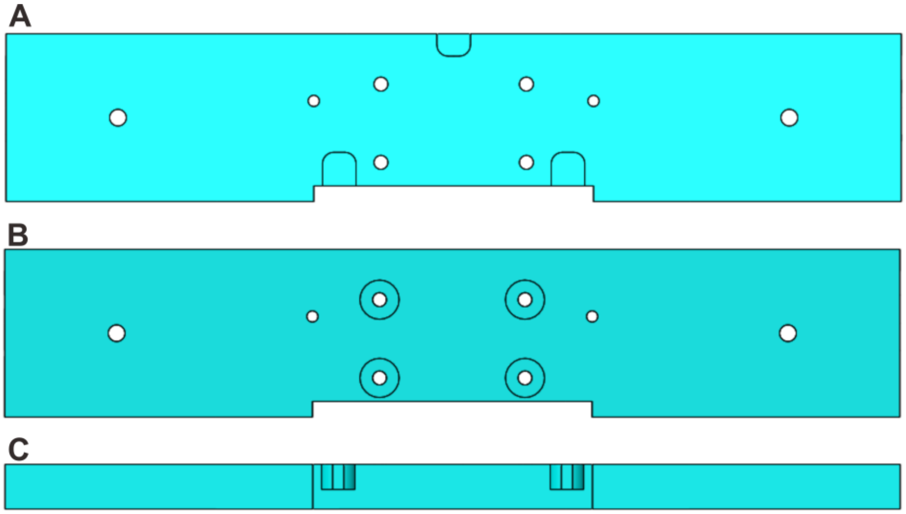

Supplemental Fig. 2 An assisting adapter designed for fixation of the window chamber during the positron imaging and the microscopic imaging: (A) the upside of the assisting adapter; (B) the downside of the assisting adapter; (C) the side view (bottom) of the assisting adapter.

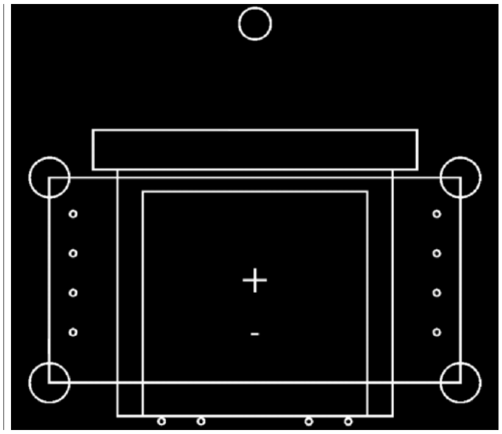

Supplemental Fig. 3 The reference pattern (transparent plastic plate) for localizing the field-of-view of microscopic imaging on the window chamber. The cross and minus mark help localize the FOV of a 1.25× objective lens of the fluorescence microscopy (7.16 mm × 5.82 mm) in the window chamber, also was used for co-registrations; the lower four black circles are used for mounting to the window chamber and are further used for fiducial markers for imaging co-registration.

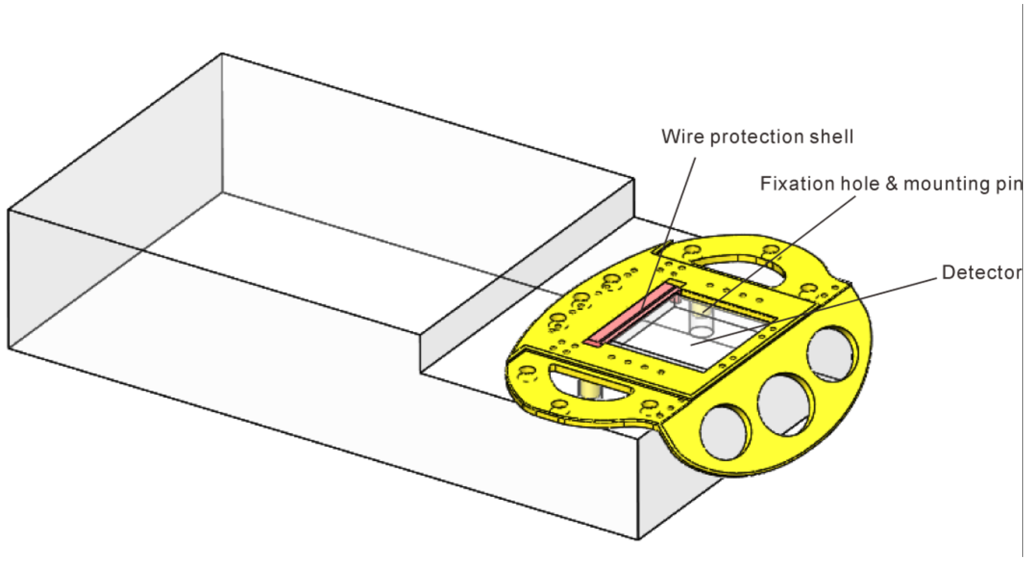

Supplemental Fig. 4 The positron camera was mounted to the glass side of the window chamber. Before the positron imaging, the cover slide of the window chamber was removed and a layer of Mylar was placed on top of the tissue. As shown in the figure, the positron camera was mounted onto the tissue covered by Mylar (where the glass cover has been removed), the detector of the positron camera was connected with the tumor tissue in the open window via the Mylar. We tried to slightly press the tissue from the skin side to improve the contact with the detector surface during the imaging.
